# Inferring Causal Factors of Core Affect Dynamics on Social Participation through the Lens of the Observer

**DOI:** 10.3390/s23062885

**Published:** 2023-03-07

**Authors:** Alessandro D’Amelio, Sabrina Patania, Sathya Buršić, Vittorio Cuculo, Giuseppe Boccignone

**Affiliations:** 1 PHuSe Lab, Department of Computer Science, University of Milano Statale, Via Celoria 18, 20133 Milan, Italy; 2 Department of Psychology, University of Milano-Bicocca, Piazza dell’Ateneo Nuovo 1, 20126 Milan, Italy

**Keywords:** affective computing, social perception, causal inference, stochastic processes, Bayesian inference

## Abstract

A core endeavour in current affective computing and social signal processing research is the construction of datasets embedding suitable ground truths to foster machine learning methods. This practice brings up hitherto overlooked intricacies. In this paper, we consider causal factors potentially arising when human raters evaluate the affect fluctuations of subjects involved in dyadic interactions and subsequently categorise them in terms of social participation traits. To gauge such factors, we propose an emulator as a statistical approximation of the human rater, and we first discuss the motivations and the rationale behind the approach.The emulator is laid down in the next section as a phenomenological model where the core affect stochastic dynamics as perceived by the rater are captured through an Ornstein–Uhlenbeck process; its parameters are then exploited to infer potential causal effects in the attribution of social traits. Following that, by resorting to a publicly available dataset, the adequacy of the model is evaluated in terms of both human raters’ emulation and machine learning predictive capabilities. We then present the results, which are followed by a general discussion concerning findings and their implications, together with advantages and potential applications of the approach.

## 1. Introduction

**Figure 1:**
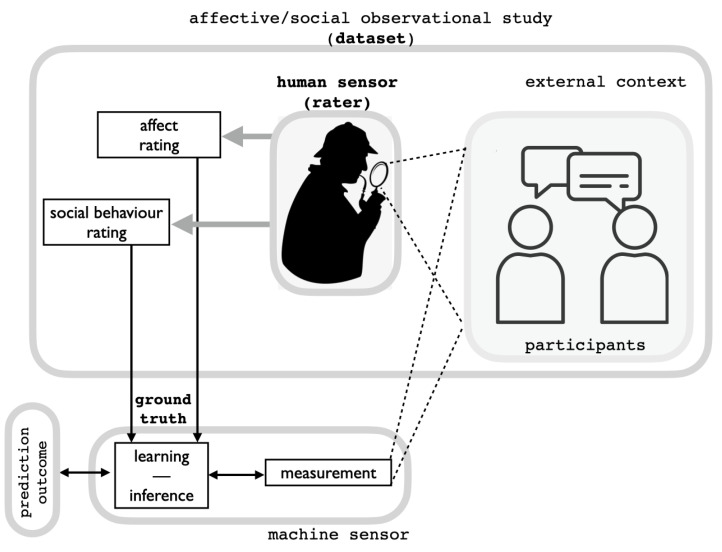
A standard scenario for dataset construction and exploitation. Multimodal data are collected from the behaviour of participants involved in some affective/social task or context (for simplicity, the recording apparatus is not explicitly shown in the scheme). A “ground truth” is provided by the rater sensing affect/social behaviour unfolding (e.g., in terms of valence and arousal dimensions). Data and ground truths are subsequently exploited for computational model training and testing to obtain prediction outcomes of interest (e.g., classification of affective/social behaviour patterns).

*The problem: context and importance.* Consider the scenario in Figure 1, a canonical one in affective computing [1,2,3]. Participants are involved in an interaction task (here, we will consider a dyadic one), while a third person (the rater or “labeller”) observes their behaviour and rates both the evolution of their affective experience and subsequently its social behaviour. Data from participants are sensed through multimodal measurements (video, audio, physiological, etc.) and ratings provided by the human sensor/rater are collected to be used as a “ground truth” for subsequent analyses. The data gathered from participants and ground truth labellings together form a dataset, which is usually exploited by machine sensing and learning procedures to achieve some prediction of interest.

Computational methods devoted to understanding and predicting social behaviour and personality traits have recently witnessed a flourish of investigations. By and large, the proposed approaches rely on solutions apt at dealing with multimodal information (e.g., audio, RGB(-D) videos, physiological information, eye movement data, etc.) in order to recognize (in the sense of the pattern recognition paradigm) psychological motifs such as personality traits [4,5,6], social behaviour [7,8,9,10,11,12], or even competence for a job [13,14,15] (but see [16] for a review). These methods typically rely on publicly available datasets to learn models and to benchmark their prediction outcomes. In brief, they are operationalised in the canonical scenario outlined in Figure 1.

**Figure 2:**
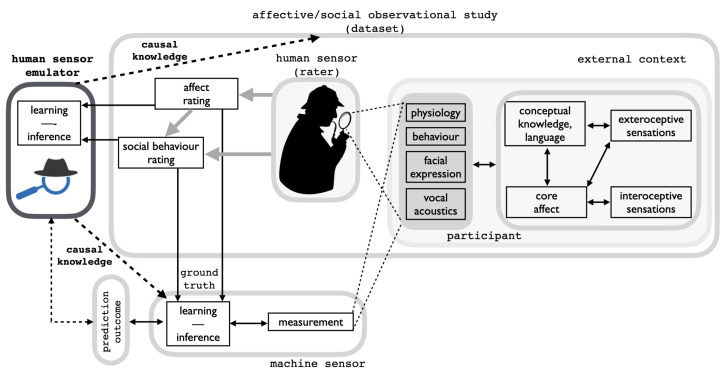
Rater’s emulation in context. The scheme draws on the scenario presented in Figure 1 while highlighting some intricacies. The participant’s behaviour (affective, social) is summarised through a variety of measurements concerning either perceivable cues (e.g., audio, video) and the accompanying physiological responses (e.g., galvanic skin response). On the one hand, the appropriate use of such measurements depends on the measurement model, which in turn entails making specific assumptions on how a participant’s affective and social behaviour are generated [17,18]; here, a psychological constructionist model is assumed (see Section 2). On the other hand, the human sensor is not a passive one, but he/she actively scrutinises participants by relying upon prior conceptual knowledge, language and biases to organize the sensory input. Subsequent ratings, e.g., of affect and social behaviour, can bring about causal dependencies (light gray arrow) between rating stages. The emulation of the rater provides a statistical approximation to the rating process suitable to unveil causal factors impinging on the labelling. The emulator’s results might be exploited to provide complementary information (dashed arrows) either to the corpus itself or to subsequent analyses performed via machine learning techniques.

However, this deceptively straightforward setting hides a complex state of affairs that should be plainly set forth from the very beginning and which is best represented in Figure 2 (but see the Discussion in Section 2).

In the case of affective/social behaviour, unfortunately, we are far from the standard conditions that we encounter, for instance, in computer vision, where classes of objects and events (e.g., actions) of interest can be objectively sensed and categorized (e.g., by assigning a “label”). Measuring general affective and more specific emotional changes or judging social behaviour is complex and fraught with difficulties. This caveat should always be taken seriously, as scrutinized in depth in terms of validity by [17,18,19]. Indeed, the “measurement model” is strongly dependent on the psychological construct adopted to frame affective/social behaviour [17].

Beyond concerns with the validity/reliability of the study behind the corpus construction and the collected data, other issues are to be considered among which the “ground truth problem” [19,20] is a prominent one. This is an apparently crude technical issue and hitherto overlooked in the affective computing literature. As a matter of fact, even in the case the raters are professionally trained (e.g., psychologists), nothing grants a reliable ground truth of the inner state of the observed subjects to be eventually produced [19]. Moreover, the adoption of folk category labels for organizing and rating patterns of affective behaviour has been questioned [20]. Furthermore, when multiple ratings are provided that concern different aspects of participants’ behaviours, these might be intertwined to some extent due to the rater’s inferential procedures. Their joint exploitation in the service of subsequent (machine learning) analyses might turn such circumstances into either a pitfall or an opportunity [20].

Clearly, the aims of the above mentioned studies (social behaviour quantification/identification) and their methodology (the classic computer vision or pattern recognition pipeline, extraction of unimodal or multimodal features followed by regression/classification) overlook or do not address per se the questions we are confronting here.

*Research plan*. To shed light on such problem, we consider the case of affect and social ratings of participants involved in dyadic interactions. Specifically, we question how the rater’s sensing of given affective dynamics impinges on his/her attribution of a particular social participation trait.

Our chief concern is in devising a suitable modelling approach to unveil the causal relationships between the affect dynamics and social trait attribution through the lens adopted by the rater. We spell the proposed model in the shape of an emulator of the rater. As opposed to a simulator that aims at modelling in detail a process, an emulator is a statistical approximation of the process under scrutiny, which allows for simpler computations than using the process simulator [21]. In our case, the approach is laid down as follows:

1. Based on the available affect annotations in the valence-arousal space and building on some preliminary work [22], we phenomenologically model core affect dynamics, a stochastic trajectory, as an Ornstein–Uhlenbeck (OU) process and identify its relevant parameters; these can be considered as descriptors of the individual’s affect tendency;
2. We then gauge the cause/effect relationship between such dynamics and social personality, its extent and the direction of such effect. To such end, we use the deconfounder method [23]. The method replaces potential unobserved confounders with an inferred latent variable; the latter is subsequently used to perform causal inference on social participation labels.

The operalisation of the approach is summarised at a glance in Figure 2. The introduction of the emulator model brings up three questions at least:

1. Is the emulated core affect dynamics consistent with the participant’s affect dynamics as perceived by the human rater? (RQ1)
2. Is the emulated social behaviour judgement causally related to the affect dynamics and reliable with respect to the human rater evaluation? (RQ2)
3. Are the emulator results to some extent suitable to be exploited in subsequent machine-oriented analyses? (RQ3)

*Method*. To address such questions, we exploit the well-known and publicly available RECOLA dataset [24]. This corpus incorporates a variety of affective and behavioural data collected along dyadic interactions where pairs of participants were asked to perform a cooperative task. In our analysis, we consider the ratings concerning the core affect state (continuous) and social personality traits (discrete), which were assigned by human raters while scrutinizing the recorded dyadic interactions. Appropriate techniques in the frameworks of Bayesian inference/prediction, nonlinear system analysis and psychometrics are exploited to operationalise the model and for its assessment.

*Novelty of the approach*. To the best of our knowledge, the present work is the first attempt in affective computing to analyse the relationship between the perceived intrapersonal affect dynamics and interpersonal behaviour from a causal standpoint by using suitable computational techniques.

Beyond the conceptual and technical novelties involved by the approach that concern both modelling and model assessment, the present proposal offers a fresh view to potentially face a number of problems in different realms of application.

The abstract gives an adequate summary of the paper.

## 2. Background and Motivation

The current practice of dataset construction for affective computing by and large considers, in the most general and interesting cases, an observational study where subjects are assigned a task in a given context. The participants’ behaviour (affective, social) is gauged through a variety of measurements concerning either perceivable cues (collected in the form of audio and video) and the related physiological responses (e.g., heart rate and galvanic skin response). Beyond questionnaires and procedures for participants’ self-assessment as to their affective state, personality traits, etc., raters are most often recruited to provide a third-person, supposedly objective “labelling” of the different facets of subjects’ behaviour. The aim is setting a ground truth suitable to be exploited by supervised or semi-supervised machine learning methods. In the discussion that follows, we assume that the observational experimental setting and data collection procedure have no flaws; however, under the above circumstances, there are issues that are seldom taken into account.

*Problems with measurements.* The subsequent appropriate use of the gathered measurements depends on the measurement model, which in turn entails making specific assumptions on how participant’s affective and social behaviour is generated [17,18]. For instance, under the Basic Emotion Theory assumption (BET [25], which by and large underpins the majority of current affective computing techniques), a stimulus (e.g., a spider) triggers a latent emotional state (e.g., fear) that results in an array of outputs (facial, physiological, etc., see Figure 2) that are strongly correlated with one another because of their common cause. In this case, measuring one observable (e.g., facial muscle movements) would suffice for emotion recognition [18]. However, recent advancements in emotion sciences [26] give evidence of a more nuanced and complex picture, which suitably accommodates within the framework of constructivist theories of emotion and social behaviour (see, for reference, [27,28]).

Given the nature of the subject, it is perhaps best we start by establishing a clear definition of the core terms exploited in what follows. In the constructive view, emotions (typically labelled by words as “fear”, “anger”, “surprise”, etc.) are considered to be abstract, ad hoc categories, where a category stands for a population of events or objects that are treated as similar because they serve a particular goal in some context [26,29].

The individual’s mental representation of a category is a concept, namely, the population of representations that correspond to a category’s events or objects. Emotions, much like other categorizations, are thus the result of situated conceptualizations. In turn, conceptual representations, the backbone of the internal representation model of the individual, are predictively tested, moment by moment, against the incoming sensory evidence—from both the external world and the body—to categorize it according to past experience (prior knowledge). This endless effort aims at anticipating the body’s needs and preparing to satisfy those needs before they arise, either by acting upon the external world/body physiology or by revising prior beliefs [30,31]. Sensory evidence is gathered through exteroception and interoception. Exteroception represents sensory changes caused by the external world; external perceptions are gathered through sight, hearing and touch or through the proprioception of self-movement and body position. Interoception denotes the sensory data that collectively describe the physiological state of the body, arising from the allostatic regulation of various bodily systems (e.g., the autonomic nervous system, the endocrine system and the immune system).

The integration of incoming exteroceptive sensations along with the internal, interoceptive information from the body gives birth to the individual’s core affective states—in simple terms, core affect. Core affect is referred to as “core” since it is grounded in the internal milieu, and the integrated sensory representation of the physiological state of the body can be described as a mental state of pleasure or displeasure, named valence, with some degree of activation or arousal [32,33,34,35]. More abstractly, valence and arousal together form a unified, continuous state-space. Emotions and core affect are thus defined at different levels, and by no means can the former be conceived as a discretised version of the latter, or vice versa the latter as a continuous representation of emotions, each emotion being a “point” in the 2D continuous space of valence and arousal—an unjustified statement which, unfortunately, is by and large assumed as a working hypothesis in the affective computing practice [1]. Cogently, the categorical nature of emotions provides the bridge between the individual’s, perceiver-independent biology of the brain and body and socially real categories that in turn allow for the sharing of emotions among individuals, i.e., the understanding by agreement of everyday concepts such as “fear” and “happiness”.

In this case, different from that postulated by BET, measures are not expected to correlate with one another, but rather together they instantiate the current emotion state. Thus, an instance of emotion can only be measured using more than one measurement modality. Furthermore, in a given context, the participant’s exteroceptive and interoceptive sensations, core affect and conceptual knowledge supported by language (see, for reference, Figure 2) work together to produce an emergent state that might be measured as a discrete emotion. In a given instance of emotion (e.g., fear of a spider), the constellation of measures will take one pattern and, in another instance (e.g., fear of public speaking), it will shift to a different pattern. All in all, this bears profound consequences both for the observational study and for the design of machine learning algorithms together with the benchmarking of their prediction outcomes.

*Problems with the rater*. The human sensor (see, for reference, Figure 2) is not a passive one but is made “of the same stuff” participants are made of. The rater actively scrutinises participants by relying upon prior conceptual knowledge, language and biases to organize the sensory input (his/her own bodily sensations and the observed participant behaviour). Emotions and social behaviours are relationship acts that unfold over time nurtured by bonding and social relations [27,28]. Interestingly enough, there is behavioural and neural evidence that even trait inference/attribution can be culture-specific [36].

The rater, rather than being engaged in passive sensing, is most likely part of a communicative act in the pragmatic sense where both verbal and nonverbal behaviour play a fundamental role [37,38,39]. A vast amount of evidence reveals that the communicative features of emotions, namely, related overt expressions, have a pivotal role in maintaining social relationships (affiliation functions) and establish a social position relative to others (social distancing function) [40,41,42]. Indeed, beyond the explicit content, the message encoded in a communicative behaviour encompasses valuable information on thoughts, desires, motivations and feelings of the sender, which may represent significant clues of the expresser’s personality. According to neurodevelopmental theories, personality can be defined as the emergent property of the individual’s continually changing, neurologically based, internal model of self in relation with the environment that dynamically integrates environmental information, both physical and social [43]. In other terms, the individual’s peculiar functioning model of the external world allows the organism to make decisions that translate into behaviours. An individual’s emotions, and their affective physiological/behavioural cascade, are nothing but a part, a primitive of such a model [44].

The judgement of a subject’s personality or social traits as performed by a rater while observing the subject’s behavioural dynamics is thus a complex inference. The observer relies on his/her own social-cognitive mechanisms (again, the internal model: goals, beliefs, values, scripts, life stories, etc.) to eventually categorize an appropriate trait label for the surmised behavioural state of the subject. Effects are well documented within the field. For example, when affective/emotional behavioural states are observed, it has been shown that smiling people are more likely to be associated with positive personality traits such as self-confidence [45], high affiliation and dominance. In addition, anger increases dominance and competence perception, whereas sadness has the opposite effect [46]. By analogy with computational pragmatics, we are under the circumstances where speakers (the participants) and listeners (the rater) use social reasoning to go beyond the literal meanings of words in order to interpret language in context; this entails that the listener, while “hearing the utterance” (observing behaviour) engages in a recursive inference on the communicative intentions of the speaker based on common ground and shared intentionality [37,38,47]. All this involves the intertwining of many primitives such as the conceptualization of exteroceptive and interoceptive sensations, with respect to a context while focusing on a specific event/object within the context, executive control to pursue the goal behind the speaker’s own communicative intentions framed by the evaluation of listener expectation/intentions (theory of mind), motor control of speech production and accompanying gestures/body behaviour [39]. For example, even the “simple” labelling of participant’s valence/arousal dynamics is likely to involve the enactment of a simulation in the rater of a participant’s core affect fluctuations in such given context [48]. Clearly, the pragmatic requirement of a shared common ground in terms of social context and culture paves the way to a conformity of judgement. However, in the meantime, it sets limits, most often overlooked in the affective computing endeavour, to the “in-the-wild” labelling practice (e.g., Western observers’ rating affect from YouTube videos where observed subjects might belong to Eastern cultures), [28,49,50].

The above considerations motivate the present study where we are specifically addressing the rater problem. In particular, we consider the case where subsequent ratings provide an evaluation of participants’ core affect in continuous form (valence and arousal trajectories unfolding in time) and the discrete labelling of their social participation traits that emerge while they are accomplishing in dyads a collaborative task.

In this perspective, there is evidence that an individual’s trends in core affect dynamics and social behaviour are intertwined to some extent, which is not a surprise in the light of a constructivist view [51]. For instance, Timmermans et al. [52] investigated the relation between intraindividual variability in core affect, interpersonal behaviour and personality traits with an experience sampling study. Their results attest to significant reciprocal influences between the mean and variability of affective status in terms of valence and arousal and interpersonal behaviour. Furthermore, results are consistent with previous studies, indicating, for instance, that highly neurotic individuals have in general lower pleasure scores [53], while individuals in roles of power seem to experience more arousal [54,55], and high core affect variability may signal insecurity and low self-esteem [56].

Under the circumstances previously outlined, we thus expect that causal dependencies are likely to occur when the two considered ratings are performed (light gray arrow in Figure 2). At the most general level, this kind of analysis should entail a process model (e.g., [31,39,48,57,58,59]), a simulator of the rater to investigate the processes that actually take place in the rating endeavour. However, one such approach brings forth a number of complex issues that at the stage of the research in the field are far from being solved. A viable solution, as put forward in Section 1, is rather to resort to a phenomenological model of the rater, an emulator supporting a statistical approximation to the rating process suitable to unveil causal factors within the labelling.

As to the research questions laid down in Section 1, on the one hand, we expect the emulator behaviour to be consistent with that of the rater. On the other hand, referring to the machine learning perspective, we hypothesise that, to the extent that we can support a causal explanation and rule out competing explanations, the likelihood of generalization is increased.

Overall, a causal explanation, by distinguishing the active from the inert components of the reasoning process behind the labelling, can provide an understanding of the processes underlying the case we are considering here. As such, it is suitable to be exploited for providing complementary information (dashed arrows in Figure 2) either to the corpus itself or to subsequent analyses performed via machine learning techniques.

## 3. Overview of the Approach

To introduce the model while keeping things simple, the computational problem at hand boils down to devising a mapping, say $p^{s}\mapsto z_{i}$, between a descriptor $p^{s}$, a vector of features/parameters suitable for capturing the affective behaviour of subject *s*, and an outcome variable/label $z_{i}$, which denotes the *i*-th trait that characterises the subject’s social behaviour. A baseline example of one such mapping can be given in terms of regression, where the $p^{s}$ components are employed as covariates,

(1) $$E\left[z_{i}^{s}|p^{s}\right]=p^{s}\eta_{i}+\epsilon ,\;\epsilon ∼N\left(0,\sigma^{2}\right)$$

and the $\eta_{i}$ is the vector of weights to be estimated; ϵ stands for noise sampled from the zero-mean normal distribution $N(0,\sigma^{2})$; E[·] denotes the expectation.

Albeit simple, such mapping suffices to conceptually capture the mapping process that, on the one hand, is assumed at the labelling level; on the other hand, it lies at the heart of the majority of affective methods. In the latter case, and typically in current end-to-end models [1], the $p^{s}$ might represent features extracted from multimodal data, and the mapping function (regression/classification) is shaped in the form of some complex architecture, e.g., a deep neural network (which generalises Equation (1) to a nonlinear mapping $z_{i}^{s}=f\left(p^{s},\epsilon \right)$).

In either case, the underlying assumption is that of a statistical correlation between affect and social traits. The model we lay down in what follows takes a different stance by addressing potential causal relations between the two, markedly at the labelling level. To such end, we first introduce a phenomenological state-space model of core affect dynamics (Section 3.1, so that model identification (the inference of model parameters) allows the computation of the $p^{s}$ descriptor; here, we draw on a preliminary study presented in [22]. Then, we unveil the causal mapping $p^{s}\mapsto z_{i}$ in terms of multiple causal inference suitable for coping with observational data (Section 3.2).

We proceed now to discuss the mathematical formalism needed in order to carry out the outlined program.

### 3.1. Observed Affect Dynamics: Modelling and Identification

At a phenomenological level, we can figure core affect dynamics as evolving from the activity of a complex open system which is conceived as subject to stochastic variations. These are the result of the entanglement of all such internal/external activities (processes at the neurobiological level) [60]. As observed from the sampling of experiential data, core affect can be unfolded in time and hence represented as a 2D *V*/*A* trajectory, i.e., a realisation of a stochastic process. Such random evolution reveals the subject-specific distinctive patterns of affective variation disclosed by the time-varying V/A levels [60]. The core affect trajectories evolving dynamically in the 2D manifold of valence and arousal can be described formally as follows.

Denote $S=\{S_{t},0\leq t\leq T\}$ and $Y=\{Y_{t},0\leq t\leq T\}$ as a pair of stochastic processes defined over $R_{s}$ and $R_{y}$, respectively.

Assume *S* to be a Markov process with an infinitesimal generator; the state-space equations describing a dynamical stochastic system can be laid down as Itô Stochastic Differential Equations (SDE, to be interpreted as an Itô stochastic integral):

(2) $$\begin{array}{cc} dS_{t} & =f\left(S_{t},U_{t}\right)dt+D^{1/2}dW_{t},\;\;\; \end{array}$$

(3) $$\begin{array}{cc} dY_{t} & =g\left(S_{t},U_{t}\right)dt+R^{1/2}dV_{t}. \end{array}$$

Here, *f* and *g* are two vector-valued functions, which are potentially time-varying; $W=\{W_{t},0\leq t\leq T\}$ and $V=\{V_{t},0\leq t\leq T\}$ represent independent Wiener processes having the same dimensions of *S* and *Y*, respectively. *D* and *R* represent diffusion coefficients that could be generally defined as a function of the states, for instance: $D=D\left(S_{t}\right),R=R\left(S_{t}\right)$.

For the sake of generality, the variable *U* is also employed as a stochastic process $U=\{U_{t},0\leq t\leq T\}$; however, simpler definitions (e.g., constant, deterministic) are allowed. *U* represents the system control (also referred to as source or input). A variety of definitions are allowed; for instance, it can be a function of *S* and *Y* (for handling feedback control) or depend on an exogenous input (e.g., the labelling sequence provided along a supervised learning stage).

Equations (2) and (3) can be easily recognised as diffusion processes, *f* and *g* being their respective drifts [61]. These processes can be thought of as the limit of the following discrete-time processes:

(4) $$\begin{array}{cc} S_{t+\Delta t}-S_{t} & =f\left(S_{t},U_{t}\right)\Delta t+D^{1/2}\sqrt{\Delta t}\epsilon_{S_{t}},\;\;\; \end{array}$$

(5) $$\begin{array}{cc} Y_{t+\Delta t}-Y_{t} & =g\left(S_{t},U_{t}\right)\Delta t+R^{1/2}\sqrt{\Delta t}\epsilon_{Y_{t}}. \end{array}$$

Equations (4) and (5) represent the Euler–Maruyama discretization of Equations (2) and (3), respectively.

Notice that Equations (2) and (3) describe a generalised input–output state-space model (SSM). Thus, *S* can be read as the latent state process; its evolution is not directly observed but can be inferred through the noisy observation process *Y*. In other terms, the states $S_{t}$ mediate the influence of the input on the output and confer memory to the system. The state and observation fluctuations are provided by noise terms $\epsilon_{S},\epsilon_{Y}$, which can be defined via the stochastic integrals $W_{t}=\int_{0}^{t}\epsilon_{S_{\tau }}d\tau$, $V_{t}=\int_{0}^{t}\epsilon_{Y_{\tau }}d\tau$.

Due to the fact that *W* and *V* denote Wiener processes, the noise components $\epsilon_{S}$ and $\epsilon_{Y}$ are specified by Gaussian additive noise, their dimension being equal to that of *S* and *Y*, respectively. Clearly, in this case, the classic diffusion process is obtained.

The typical input–output SSM can be recovered from Equations (2) and (3), under the conditional independence assumption of current observation $Y_{t}$ on the previous one $Y_{t-1}$, given $S_{t}$,

$$Y_{t}⊥Y_{t-1}|S_{t};$$

namely,

(6) $$\begin{array}{cc} dS_{t} & =f\left(S_{t},U_{t}\right)dt+D^{1/2}dW_{t},\;\;\; \end{array}$$

(7) $$\begin{array}{cc} Y_{t} & =g\left(S_{t},U_{t}\right)+R^{1/2}\epsilon_{Y_{t}}. \end{array}$$

By setting $f\left(S_{t},U_{t}\right)=B\left(U-S_{t}\right)$ and $g\left(S_{t},U_{t}\right)=S_{t}$ in Equations (6) and (7), the following model of core affect’s stochastic dynamics is eventually recovered:

(8) $$\begin{array}{cc} dS_{t} & =B\left(U-S_{t}\right)dt+D^{1/2}dW_{t},\;\;\; \end{array}$$

(9) $$\begin{array}{cc} Y_{t} & =S_{t}+R^{1/2}\epsilon_{Y_{t}}, \end{array}$$

In Equation (8), *B* is a positive definite square matrix; in order to ensure the stability of the process (convergence to the stationary distribution), the matrix *B* is required to have all positive eigenvalues [62]. The *U* parameter can be considered as a constant (U=const), or time-varying in the most general case. The instantaneous change in $S_{t}$ ($dS_{t}$) depends on the relative distance of the current state $S_{t}$ being from the point *U*. The latter is typically called a steady state or attractor. The state Equation (8) can be easily recognised as a form of the Ornstein–Uhlenbeck (OU) process [63]; hence, Equations (8) and (9) together represent an Ornstein–Uhlenbeck State Space Model (OU-SSM).

This theoretical model for the description of covert affect fluctuations has been adopted by [64], showing that differences between subjects are reflected in variations of model’s parameters. In a nutshell, the evolution over time of a trajectory describing the moment-to-moment covert affective state of a subject can be thought of as generated from Equations (8) and (9) that stochastically evolve in a 2D space, whose axes represent valence and arousal, respectively. Crucially, albeit evolving randomly, such affective paths retain individual differences that are eventually captured by the SDE parameters.

Under such circumstances, the relevant parameters of the OU-SSM can be interpreted as follows:

- *B*: the parameter controls the strength of the “attraction” towards *U*. For a two-dimensional trajectory, it is defined as a 2×2 matrix:
  (10) $$B=\left[\begin{array}{cc} B_{AA} & B_{AV}\\ B_{VA} & B_{VV} \end{array}\right]$$
  $B_{AA}$ and $B_{VV}$ represent the drift of the process towards the steady state *U* in the arousal and valence dimension, respectively. The off-diagonal elements $B_{AV}=B_{VA}=\rho_{B}\sqrt{B_{AA}B_{VV}}$ describe the cross-correlation between drift in both dimensions. Higher values of $B_{AA}$ or $B_{VV}$ will magnify the difference between the actual state and $U_{A}$ or $U_{V}$, respectively; as a result, this will produce a faster change towards *U* for that specific dimension. For high cross-correlation values, increasing value of the drift in one dimension will produce increasing values on the other. This will cause more curved trajectories towards the attractor *U*. For these reasons, the parameter *B* is often referred to as the dampening force or centralising tendency. It is surmised that the strength of this force reflects the regulatory processes devised to keep a person’s core affect under control.
- *U*: for a two-dimensional trajectory, this parameter has the shape of a 2D vector. Intuitively, it operates as an anchor describing the baseline emotional behaviour of a subject, an affective “home base” or comfort zone of an individual. By constantly pulling core affect back to its home base, the attractor keeps the system in balance, creating an emergent coherence around it;
- *D*: this parameter denotes a 2×2 correlation matrix controlling the variances and covariances of the 2 driving white noise processes $dW_{t}$. Higher values of variances/covariances will produce noisier/more anisotropic core affect trajectories.

In brief, the affective home base, attractor strength and variability can be considered as the key mechanisms governing the countless number of ways people can display changes and fluctuations in their core affect [60].

As said, the affect dynamics—and its driving parameters—is an individual-based dynamics. Thus, denote $\Theta^{s}$ as the core affect parameters defining the OU-SSM related to subject *s*.

By exploiting the Euler–Maruyama discretisation scheme (Equations (4) and (5)) and relying on the Markov property germane to the OU process, the probability of the observed trajectory, given the parameters $\Theta^{s}=\left\{U^{s},B^{s},D^{s},R^{s}\right\}$, is:

(11) $$P\left(Y^{s}|\Theta^{s}\right)=\prod_{t=1}^{N-1}P\left(Y_{t+\Delta_{t}}^{s}|Y_{t}^{s},\Theta \right).$$

The posterior distribution of the OU parameters given the sample trajectory is obtained by inverting probabilities via Bayes’ theorem:

(12) $$P\left(\Theta^{s}|Y^{s}\right)=\frac{P\left(Y^{s}|\Theta^{s}\right)P\left(\Theta^{s}\right)}{P(Y^{s})}.$$

Given the inferred posterior distribution, a concise description of the individual’s core affect dynamics can be derived as follows. Each posterior distribution associated with each parameter in the state equation of the OU-SSM (Equation (2)) is summarized via its posterior sample mean; this provides the distribution summaries related to the diagonal and one of the off-diagonal components of the $B^{s}$ and $D^{s}$ matrices (recall that $B^{s}$ and $D^{s}$ are symmetric matrices) and the two average values obtained from the 2D $U^{s}$ vector.

A descriptor $p^{s}$ is obtained by joining the inferred OU parameters for the *s*-th subject, which writes in vector form

(13) $$p^{s}=\left[{\hat{U}}_{A}^{s},{\hat{U}}_{V}^{s},{\hat{B}}_{AA}^{s},{\hat{B}}_{AV}^{s},{\hat{B}}_{VV}^{s},{\hat{D}}_{AA}^{s},{\hat{D}}_{AV}^{s},{\hat{D}}_{VV}^{s}\right].$$

Eventually, the vector $p^{s}\in R^{8}$ compactly represents the core affect dynamics for the *s*-th subject.

### 3.2. From Observed Affect Dynamics to Social Participation Labelling: Unveiling Causal Effects

We now consider the vector $p^{s}$ as the set of potential causes eventually conditioning the outcome $z_{i}^{s}$. In a multiple causal inference approach, the inferred OU-SSM parameters represent the ensemble of treatments that the rater receives, and the problem is to quantify their effect on the attribution of a given level of social participation.

Clearly, a correlational mapping $p^{s}\mapsto z_{i}$, e.g., the one represented in Equation (1), does not allow per se to infer a causal effect. Cogently, causal inference requires to analyse the mapping under active intervention (**do**) but relying solely on data collected from the observational study. In Pearl’s [65] notation,

$$E[z_{i}^{s}|\mathrm{do}\left(p^{s}\right)].$$

The difference is subtle but fundamental; a properly defined causal model allows for answering counterfactual questions such as: “What would have been the rated $z_{i}$ value, had the subject exhibited a certain affective behaviour?”

In general [66]:

(14) $$E\left[z_{i}^{s}|p^{s}\right]\neq E\left[z_{i}^{s}|\mathrm{do}\left(p^{s}\right)\right]$$

The reason why this happens is due to the fact that the data may hide some confounders i.e., variables that affect both the causes (emotional dynamics) and effect (social traits). The general and most effective solution to the problem of causal inference is the use of randomization in the study design phase. On the contrary, when relying on purely observational data, the general solution is to spot and measure a sufficient number of confounding variables $c^{s}=\left(c_{1}^{s},...,c_{K}^{s}\right)$ and adjusting for it such that $p^{s}$ and $z_{i}^{s}$ are conditionally independent given $c^{s}$, $p^{s}⊥z_{i}^{s}|c^{s}$.

The observation of all confounding variables renders the causes (affect dynamics) conditionally independent of the outcome (social behaviour). In this case, we can say that ignorability holds, hence the causal effect can be identified from observational data [65]:

(15) $$E\left[z_{i}^{s}|\mathrm{do}\left(p^{s}\right)\right]=E\left[E\left[z_{i}^{s}|p^{s},c^{s}\right]\right]$$

What are the factors that if measured may ensure ignorability? Both V/A trajectories and social participation labels are the result of the annotation of raters. As discussed from the beginning, these annotations are subjective attributions of each rater based on what subjects express through facial expression, prosody, gestures, adopted vocabulary, subject’s cultural origin or maybe some form of the rater’s instinctively perceived sympathy/dislike for a subject. It seems reasonable to assume that at least a subset of this factors may have an influence on both the rating of V/A and social traits. All such variables may be confounders that when left unobserved produce association between perceived emotional dynamics and social attitude. Notice that, although few of these variables may be measured with relative ease (e.g., facial expression, prosody, gesture), others are somewhat difficult or impossible to estimate (e.g., perceived like/dislike). A striking example is provided by the role of perceived personality; from the social cognitive point of view, information inferred from facial expressions or auditory behaviours could result in the attribution of personality traits. Even very short movies depicting social behaviours lead the perceivers to attribute personality traits to the observed actors [67]. In such a setting, the rater’s inferred personalty may act as a confounder impinging on both V/A rating and social participation attribution.

**Figure 3:**
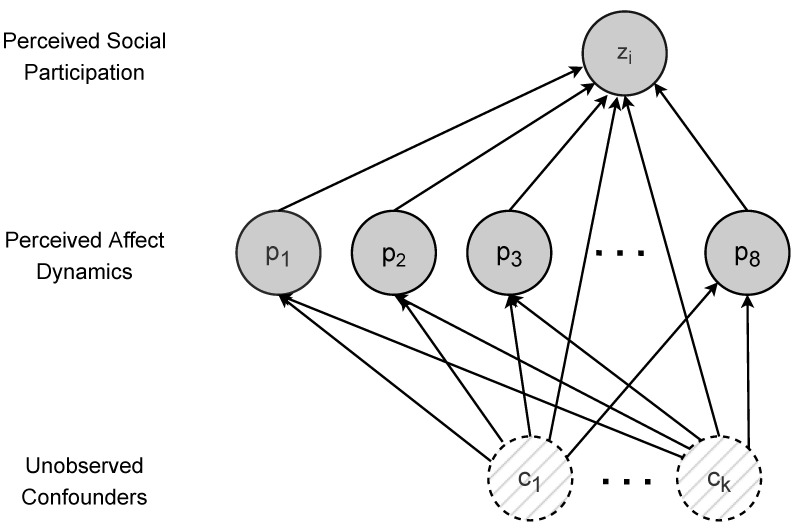
The assumed causal graph. The apparent (perceived) social behaviour is caused by the many variables describing affect dynamics. Both, though, may be subject to a set of unobserved common causes (confounders).

It is known that performing causal inference from observational data requires making assumptions on how such data may have been generated. These assumptions can be made explicit through a causal graph, encoding the causal relationships between the relevant variables in the form of a directed acyclic graph (DAG). The causal graph assumed in this work is depicted in Figure 3.

In order to infer the causality of $p^{s}$ on $z_{i}^{s}$ it is therefore necessary to spot and measure a sufficient number of such confounding variables, thus ensuring ignorability. This is a standard yet uncheckable assumption.

Recently, Wang and Blei [23] proposed the deconfounder, an algorithm that combines unsupervised machine learning and predictive model checking to perform causal inference in multiple-cause settings. The method is built upon three steps:

1. Fit a good factor model of assigned causes able to capture the joint distribution $P(c^{s},p_{1}^{s},...,p_{L}^{s})$, where $c^{s}$ is a local factor;
2. Use the model to infer the latent variable for each individual $P(c^{s}|p_{1}^{s},...,p_{L}^{s})$;
3. Perform causal inference by fitting an outcome model adjusted for confounding by conditioning on the inferred factor model latent variables $c^{s}$.

If the factor model is able to capture the joint distribution of the causes $P(p_{1}^{s},...,p_{L}^{s})$, then all the causes are conditionally independent given the latent factor $c^{s}$:

(16) $$P\left(p_{1}^{s},\ldots ,p_{L}^{s}|c^{s}\right)=\prod_{j=1}^{L}P\left(p_{j}^{s}|c^{s}\right)$$

Intuitively, a well specified factor model captures the observed dependencies among the causes which should contain information about some of the confounders (e.g., perceived personality or like/dislike). Consequently, the local factors that are employed as substitute confounders could eventually deliver information about confounders if this is encoded in the dependency structure of the data.

The deconfounder relies on few fundamental but reasonable assumptions. In addition to the SUTVA (Stable Unit Treatment Values Assumption, SUTVA is really two assumptions rolled into one: (1) the potential outcomes for any unit do not vary with the treatments assigned to other units; (2) for each unit, there are no different forms or versions of each treatment level, which lead to different potential outcomes) [68], and it is required that there are no single-cause confounders, i.e., variables that have an effect on both the outcome and only one of the treatments. If we assume that there are no unobserved variables that impinge on the social participation attribution and one of the factors defining affect dynamics, then Equation (16) implies ignorability: $p^{s}⊥z_{i}^{s}|c^{s}$ [23]. Note that this assumption (sometimes called weak unconfoundedness) is weaker than assuming no unobserved (multi-cause) confounders.

Secondly, the deconfounder assumes that $p_{j}^{s}⊥p_{-j}^{s}|c^{s}$ for any j=1,2,⋯8 [69]. This assumption states that there cannot exist a causal relationship among the treatments (see Figure 3).

Following Equation (15), it is possible to perform causal inference on observational data via conditioning on confounders. Given a good factor model, we can use the expected value of the inferred posterior of the latent variable

(17) $${\hat{c}}^{s}=E\left[c^{s}|p^{s}\right]$$

as confounders; thus, Equation (15) writes:

(18) $$E\left[z_{i}^{s}|\mathrm{do}\left(p^{s}\right)\right]=E\left[E\left[z_{i}^{s}|p^{s},{\hat{c}}^{s}\right]\right],$$

which represents the causal outcome model.

## 4. Methods

The approach described in the previous section has been employed in the analysis of observational data collected in a publicly available dataset, as commonly carried out in the affective computing and machine learning realms.

Specifically, we have chosen the well-known and widely used multimodal RECOLA (REmote COLlaborative and Affective interactions) corpus [24]. This dataset collects observational data from participants’ spontaneous dyadic interactions; furthermore, it provides continuously rated core affect (valence and arousal) dynamics and social participation evaluation obtained from third-person annotators. For the reader’s convenience, in what follows, we give a bare recap of its main characteristics relevant for this study, while leaving the details to [24].

### 4.1. Participants

For constructing the corpus, 46 participants (27 females, 19 males, mean age 22 years, standard deviation 3 years) were recruited and divided into 23 dyadic teams. All subjects were students from the Department of Psychology of the Université de Fribourg and French speaking (33 had French as the mother tongue, 8 Italian, 4 German and 1 Portuguese). Amongst the 46 participants, only 34 gave their consent to share their data to the public. Data from 23 participants (training and validation partitions) are made publicly available (and hence used in this study), whereas the other subjects (test partition) are not publicly available.

### 4.2. Apparatus

Skype was used in full-screen for the video-conference in which the dyadic collaboration task was performed. Audio data were captured by unidirectional headset microphones and recorded. Two HD webcams were used for each participant. The first webcam only captured the video data and was used for the Skype video-conference. The second webcam was used to record both audio, from the built-in omnidirectional microphone, and video with the software provided by the manufacturer. The headset microphones were placed on the head of the participants and the camera angle adjusted to have full face visibility on the screen. For the physiological data, both electro-dermal activity (EDA) and electro-cardiogram (ECG) signals were recorded. A careful synchronization of all signals was performed. Eventually, the RECOLA database includes 9.5 h of multimodal recordings.

For facilitating the remote annotation of data, a web-based annotation tool, ANNEMO, was specifically developed (and publicly released). The tool allows for a setting with one time-continuous annotation for each affective dimension. The web-browser interface displays the audiovisual recording (video) and the annotation cursor one below the other. The arousal and valence were annotated separately and time-continuously, using a slider with values ranging from −1 to +1 and a step of 0.01.

### 4.3. Procedure

The participants of a team were introduced to each other and received an introduction to the experiment. They were told that they were taking part in a study focusing on communication between people by using computer-supported tools, for an overall duration of about an hour. Each participant first received a questionnaire to evaluate his/her current emotional state by using the Self-Assessment Manikin (SAM, [70]).

After this evaluation, participants started individually solving the survival task for a maximum duration of 10 min. While the members of a team were completing the individual task, it was decided whether they would be constituting a positive or a negative group, according to the SAM self-reports. The facilitators of the experiments decided which participant would receive a positive or a negative mood induction according to the self-reported SAM’s valence. The mood induction procedure was used to increase the difference in emotional valence between participants of a team; the aim was to have a balanced distribution of team members between positive and negative moods group [71]. In the end, 12 participants had a negative mood when they started the discussion with their teammate, 24 had a neutral mood and 10 a positive one.

Participants performed in dyads remotely through video conference; they engaged in a remote discussion to solve a collaborative task (“Winter survival task”, [72]). The task was originally designed by the National Aeronautics and Space Administration (NASA) to train astronauts before the first moon landing. Participants are asked to reach consensus on how to survive in a disaster scenario. It is frequently used in social psychology for eliciting decision-making processes in small groups; the solution to the problem is not straightforward and may require an intensive discussion.

At the end of the recordings, annotation of participants’ core affect was performed (6 raters: 3 males, 3 females). Affective behaviour was continuously rated over time resorting to the two core affect dimensions of valence *V* and arousal *A*. Each annotator was instructed orally and received instructions explaining in detail the procedure to follow. Before starting the annotation of the data from the RECOLA corpus, annotators first performed the annotation of two video sequences selected from the SEMAINE corpus, to become familiar with the annotation interface.

The annotation of the social dimensions was performed after the two affective dimensions for each sequence. Social behaviour was summarised by annotators according to five participation dimensions: agreement (that is, judging whether the subject seems to agree with his/her partner), dominance (the subject appears to be dominant), engagement (the person shows to be engaged), performance (the person’s speech is clear and relevant to the task) and rapport (whether the person and his/her partner give the impression to be or that they could become friends). These basic primitives were chosen based on various studies in the literature [24]. The annotation of dimensions of social behaviours was performed using a 7-Likert scale. A careful post-processing of the annotations was conducted to reduce unwanted variabilities in the data (e.g., blanks or jumps due to re-annotation) and to provide a ground truth for the automatic recognition of the annotated behaviours.

### 4.4. Data Analysis

In this section, for each component of the emulator, we detail the Bayesian statistical procedures for instantiating and inferring the model parameters together with the measures adopted for assessment/validation.

#### 4.4.1. OU-SSM Parameters Inference and Validation

*OU-SSM inference*. For each subject $s=1,2,\cdots ,N_{s}$, $N_{s}$ being the total number of rated subjects in the RECOLA dataset, the 6 raters annotated his/her emotional state evolution as a time-continuous V/A trajectory; a single average trajectory is obtained as the result of the Evaluator Weighted Estimator (EWE) measure [73]. EWE performs a weighted average of each rater on the basis of its agreement with the others (agreement is operationally defined in terms of Pearson’s Correlation). Under the assumptions of this work, the EWE, rather than providing a kind of “objective” ground truth, is best described as a measure of social conformity of the raters in their judgement/inference of subject’s observed affective/social behaviour. Drawing on earlier, preliminary work [22], we formally consider the EWE averaged annotated V/A trajectory of the subject *s* as a realization $Y^{s}$ from Equation (9).

We estimate the set of subject-specific parameters $\Theta^{s}$ (see, for reference, Equation (12)) to the EWE averaged annotated V/A trajectories via Bayesian inference.

*OU-SSM validation*. Once the OU-SSM parameters are available, then the emulator can in theory be exploited to sample stochastic V/A trajectories to be compared with those generated by a human rating. However, such comparison, when not barely qualitative such as in [56], for example, is not readily apparent. A simple solution was devised in our preliminary study [22]. A principled solution can be found by recalling that, at their origin, V/A trajectories are the outcome of a nonlinear dynamical system that involves a large number of interacting elements or components [48] that is of a complex system. In this case, a number of methods are available [74]. One such tool is recurrence analysis (RA), a nonlinear analysis method that can be employed to scrutinise both stationary and nonstationary data [75]. The virtue of RA, by contrast with other linear time-series methods, is that it does not require assumptions about the structure of the time series being investigated or the underlying dynamics that shapes the recorded trajectory [75]. Albeit still relatively new, there is now substantial evidence that suggests it is potentially one of the most robust and generally applicable methods for assessing the dynamics of biological and human behaviour [74]. In a nutshell, RA identifies the dynamics of a system by discerning whether the states of the system behaviour recur over time and, if states are recurrent over time, the degree to which the patterning of recurrences is highly regular or repetitive. RA can also be extended to uncover the dynamic similarity, mutual influence, or coordinated structure that exists between two different behavioural time series or sequences of behavioural events. This latter form is termed cross-recurrence analysis (CRA) and can be considered as a generalisation of the linear cross-correlation function [75]. In this case, recurrent points in a cross-recurrence plot (CRP) correspond to states, events or categories in two time series or behavioural trajectories that are recurrent with each other [75].

Different measures can be derived from CRA. We use here determinism (DET), laminarity (LAM) and maximum diagonal line length (MDL). Determinism is defined as

(19) $$DET=\frac{\sum_{l=l_{min}}^{N}lP\left(l\right)}{\sum_{l=1}^{N}lP\left(l\right)}$$

where *l* stands for the lengths of diagonal lines in the CRP, and P(l) is the number of lines of length equal to *l*, while $l_{min}$ is set to 2. Intuitively, this measure quantifies the percentage of recurrence points which form diagonal lines or, in other words, how many of the individual repetitions co-occur in the two trajectories in the same order. Laminarity is defined as

(20) $$LAM=\frac{\sum_{v=v_{min}}^{N}vP\left(v\right)}{\sum_{v=1}^{N}vP\left(v\right)}$$

where, similarly to the determinism, *v* stands for the lengths of vertical lines, P(v) represents the number of vertical lines in the CRP, and $v_{mins}$ is equal to 2. Laminarity accounts for the number of recurrence points which form vertical lines, that is to say, it measures the proportion of time that the two time series are in the same state, or “laminar”, meaning that they exhibit the same behaviour or pattern at a given point in time. Finally, the maximum diagonal line length, as one is led to believe, refers to the length of the longest diagonal line in CRP. It is a measure used to quantify the duration of the longest cross-repeating trajectory, where a longer diagonal line represents a stronger coupling between the two time series.

#### 4.4.2. Causal Analysis

*Causal model determination*. The first step required by the deconfounder method is to find, fit and check a factor model of the causes. One of the most common and simple factor models is Probabilistic Principal Component Analysis (PPCA). The model is defined as follows:

(21) $$\begin{array}{c} c^{s}∼N\left(0,\lambda^{2}I\right)\\ p^{s}|c^{s}∼N\left(c^{s⊺}W,\nu^{2}I\right), \end{array}$$

where $W\in R^{K\times L}$ is the projection matrix from the *K*-dimensional latent space to the data. We fit this model using ADVI and obtain the posterior distribution over the free parameters of the model ($\nu^{2}$, W). We now have access to estimates of $P(c^{s}|p^{s})$.

In order to ensure that the learned factor model is able to capture the joint distribution of the causes, posterior predictive checks (PPC) are performed on a held-out set of data. PPC consists of first sampling values for the held-out causes from the predictive distribution of the PPCA model:

(22) $$P\left({\hat{p}}_{\;\mathrm{held}\;}^{s}|p_{\;\mathrm{obs}\;}^{s}\right)=\int p\left(p_{\;\mathrm{held}\;}^{s}|c^{s}\right)p\left(c^{s}|p_{\mathrm{obs}}^{s}\right)dc^{s}$$

where ${\hat{p}}_{\;\mathrm{held}\;}^{s}$ is the replicated held-out data. We then compute the expected held-out log-likelihood on both the replicated and real held-out data (${\hat{p}}_{\;\mathrm{held}\;}^{s}$ and $p_{\;\mathrm{held}\;}^{s}$, respectively) under the PPCA model:

(23) $$\begin{array}{c} t\left(p_{\mathrm{held}}^{s}\right)=E_{c,W}\left[\log P\left(p_{\mathrm{held}}^{s}|c^{s},W\right)|p_{\mathrm{obs}}^{s}\right]\\ t\left({\hat{p}}_{\mathrm{held}}^{s}\right)=E_{c,W}\left[\log P\left({\hat{p}}_{\mathrm{held}}^{s}|c^{s},W\right)|p_{\mathrm{obs}}^{s}\right] \end{array}$$

These values tell how likely the replicated and real data are under the learnt factor model; a good model will produce similar values of the expected held-out log-likelihoods. The predictive score (how well the model approximates the causes) is defined as:

(24) $$\;\mathrm{score}\;=P\left(t\left({\hat{p}}_{\;\mathrm{held}\;}^{s}\right)<t\left(p_{\;\mathrm{held}\;}^{s}\right)\right).$$

An ideal predictive score will have values around 0.5, while a mismatched model will produce very low scores.

After fitting and checking the PPCA model with K=2 on the assigned causes (OU parameters), the PPC produces a predictive score of 0.44; hence, we conclude that the model is able to capture the joint distribution of the causes.

The obtained factor model is employed to compute, for each subject, a local factor. The latter is used as a substitute confounder in the causal outcome model (Equation (18)). We approximate the $P(z_{i}^{s}|p^{s},{\hat{c}}^{s})$ (causal outcome model) with the linear function (Truncated BLR):

(25) $$f\left(p^{s},{\hat{c}}^{s}\right)=p^{s}\eta_{i}+{\hat{c}}^{s}\gamma_{i}+\epsilon \;\epsilon ∼\tilde{N}\left(0,\sigma_{i}^{2}\right),$$

where $\tilde{N}\left(\cdot \right)$ is a truncated Normal distribution that assigns zero probability to the samples outside the range defined by a 7-Likert scale. Computing the inner expectation on the right-hand side of Equation (18) boils down to computing the expectation of a Gaussian, while the outer expectation is approximated via Monte Carlo, i.e., averaging over the latent factor population distribution (this is sometimes referred to as back-door adjustment o *G*-formula):

(26) $$E\left[E\left[z_{i}^{s}|p^{s},{\hat{c}}^{s}\right]\right]=\frac{1}{N_{s}}\sum_{s=1}^{N_{s}}f\left(p^{s},{\hat{c}}^{s}\right)$$

The causal outcome model parameters $\eta_{i}$, $\gamma_{i}$ and $\sigma^{2}$ are fitted to the augmented dataset {($p^{s}$, $z_{i}^{s}$, ${\hat{c}}^{s}$)} via ADVI which delivers estimates for the model’s posterior distribution. The inferred regression coefficients represent the causal effect of raising the causes by one unit.

To set a baseline for non-causal inference, we employ the standard linear regression model specified in Equation (1) as well as a purely associative truncated Bayesian Linear Regression.

*Causal model validation*. The learnt causal outcome model can be employed to sample *R* model-simulated raters. More specifically, the *r*-th rater can be obtained by sampling from the posterior distribution of the outcome model’s parameters:

(27) $$\eta_{i,r}∼P\left(\eta_{i}\right)\;\gamma_{i,r}∼P\left(\gamma_{i}\right)\;\sigma_{i,r}∼P\left(\sigma_{i}\right).$$

Define the value $\mu_{i,r}^{s}=p^{s}\eta_{i,r}+{\hat{c}}^{s}\gamma_{i,r}$. The *r*-th rating is thus obtained as:

(28) $${\hat{z}}_{i,r}^{s}=\left\lfloor \tilde{N}\left(\mu_{i,r}^{s},\sigma_{i,r}^{2}\right)\right\rceil ,$$

where the ⌊·⌉ operator defines rounding to the nearest integer.

A reliable model-simulated rater should produce social trait ratings that are internally consistent as well as in agreement with those provided by human raters. In psychometrics, a variety of Inter-Rater Reliability (IRR) statistics are available in order to assess the agreement between many raters (see [76] for an overview). The intra-class correlation (ICC) is typically employed for the assessment of IRR in the presence of ordinal, interval or ratio variables (e.g., 7-Likert scale ratings) [77,78]. ICC estimates of 1 indicate perfect agreement between raters, while values approaching −1 denote systematic disagreement. In our case, the ICC is expected to quantify the amount of inter-rater agreement when confronting human raters with those simulated by the proposed model.

*Causal model predictive performance evaluation*. In order to assess the predictive performances of the proposed causal outcome model, we compute the average predictive log-likelihood (PLL) on *n* held-out subjects. The latter is defined as:

(29) $$\begin{array}{c} \mathrm{PLL}=\frac{1}{n}\sum_{j=1}^{n}E_{\eta ,\gamma }\left[\log P\left(z^{j,held}|\eta ,\gamma ,p^{j,held},{\hat{c}}^{j,held}\right)\right] \end{array}$$

PLL allows for measuring the goodness of fit for a model. The higher the value of the PLL, the better a model fits a dataset. By computing it on held-out data, we are testing the ability of the model to generalize on unseen data.

Eventually, in the vein of [23], in order to test the capabilities of the causal outcome model under intervention, the average PPL can be computed on a test set of “uncommon” subjects. Specifically, we consider the German native speaking subjects as held-out examples while leaving all the other subjects in the training set. This should change the distribution of the samples composing the test set with respect to the training set. In such cases, a causal model should produce more accurate results if compared with a purely associative one.

## 5. Results

### 5.1. OU-SSM Results

**Figure 4:**
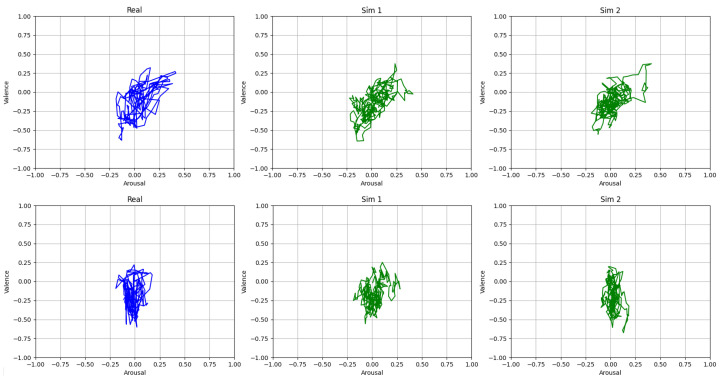
V/A trajectories from subjects P30 (**top**) and P34 (**bottom**) as annotated by human raters (in blue) and two emulated raters for each subject (in green).

To give a tangible clue as to how the OU-SSM component of the emulator operates, in Figure 4, we display a qualitative comparison between the V/A trajectories generated by the human rater (per observed subject) and those sampled by the emulator.

**Figure 5:**
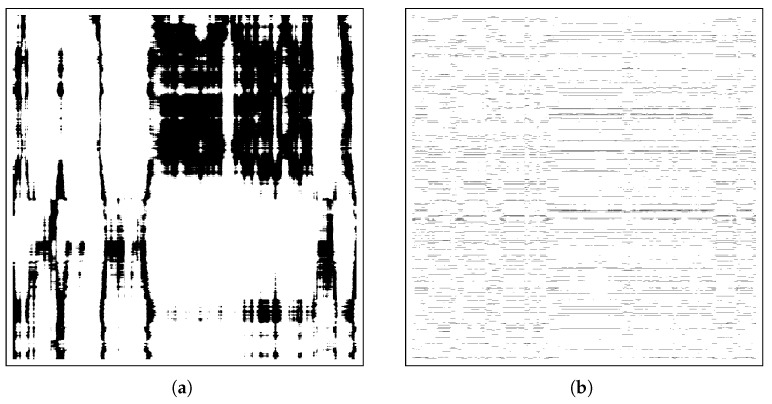
Cross recurrence plots of the trajectories from human raters compared against an emulated rater and a random rater. The *x*- and *y*-axes represent the time axes of the V/A trajectories generated by the synthetic and human rater, respectively. Each point in the matrix visualises as a binary value (black: near, white: far) the adjacency of two V/A points in the respective trajectories. Off-diagonal dots represent adjacency at different time lags. (**a**) emulated rater vs. human rater; (**b**) random rater vs. human rater.

For a quantitative assessment, Table 1 reports DET, LAM, and MDL measurements performed through cross-recurrence analysis via cross-recurrence plots. One example of CRPs underpinning the above measurements is presented in Figure 5, where the emulator performance is contrasted with that of a random trajectory generator (baseline).

#### Discussion

The examples provided in Figure 4 graphically illustrate that, on the basis of the fitted parameters, the OU-SSM component of the emulator generates/predicts V/A trajectories that closely resemble the observed human-rated trajectories in shape and dispersion (keeping the same time differences as in the observed data). It is important to stress that the replicated trajectories cannot follow exactly the same path as the observed trajectory because the model is inherently stochastic. Nonetheless, the examples in Figure 4 illustrate that the spatial characteristics of the observed trajectories are well preserved in the emulated ones.

Besides this crude visual assessment of the similarity between the observed and replicated trajectories, we also quantitatively examined the correlation between the two by resorting to measurements derived from cross-recurrence plots. As shown in Figure 5, the emerging 2D patterns are of major interest here. They represent segments on both trajectories, which run parallel for some time; the frequency and length of their appearance are related to a similarity between the dynamics of both systems; such complex patterns do not arise, as expected, when confronting human-raters’ trajectories with pure randomly generated trajectories (i.e., a trivially different physical process). DET, LAM and MDL metrics give evidence of a remarkable agreement for all participants so that it can be assumed that both data series come from the same process [75]. Note that determinism and laminarity are measures of the finer temporal structure, which entails that the OU-SSM model is effective at capturing the idiosyncratic characteristics of individual’s core affect dynamics.

Interestingly enough, these measures could be used to compare over time across different types of experimental contexts, and participants [75].

### 5.2. Causal Analysis Results

*Causal Factors.* The posterior distribution over the outcome model parameters $P(\eta_{i},\gamma_{i}|\{(p^{s}$, $z_{i}^{s}$, ${\hat{c}}^{s})\})$ allows for quantifying the uncertainty on the estimation. The latter can be summarised by considering the interval of values lying within the 95% of the estimated posterior distribution. Such quantity is called Highest Density Interval (HDI) and is widely adopted in Bayesian statistics for the assessment of statistical significance [79].

More specifically, values lying outside the HDI can be considered unlikely under the observed data and model. Hence, if the 0 value dwells outside the HDI of a given regression coefficient, then it can be “rejected” as a credible value for describing the relationship between the associated OU parameter and a given social trait dimension; consequently, we can conclude that a statistically significant linear relationship exists. Table 2 reports the regression coefficients whose posterior HDI does not include the zero value for the purely correlational Bayesian Linear Regression model. Following the same rationale, in Table 3, the regression coefficients of the causal outcome model whose HDI does not contain the 0 are reported.

*Causal model validation.*Table 4 reports the ICC values computed on RECOLA’s human raters (ICC Human), on six sampled raters from Equation (28) (ICC Model) and from the set of 12 raters obtained by joining the previous two (ICC Humans + Model). Recall that, according to ICC, inter-rater agreement can be considered poor for values <0.40, fair if 0.40<ICC<0.59, good if 0.60<ICC<0.74 and excellent for ICC values greater than 0.74 [80]. In order to check whether any difference between the IRR of male and female raters in the RECOLA dataset exists, ICC has been computed for the two groups separately; in both cases, male and female raters exhibited good agreement (0.60<ICC<0.74) with no significant differences between the two groups (independent sample *t*-test, *p*-value >0.05).

Results reveal good agreement between raters simulated from the outcome model (0.6<ICC<0.74) and excellent agreement between model-simulated and human raters as well as human raters only (0.75<ICC<1.).

*Causal model predictive performance evaluation.*Table 5 reports average PLLs on a “typical” test set constructed by randomly selecting 30% of the subjects (first row) and on a test set of uncommon subjects (German native speakers, second row). We report results for the causal outcome model and the (non-causal) truncated BLR in terms of PLL; for comparison, results for a standard Bayesian Linear Regression model (see, for reference, Equation (1)) are reported too.

**Figure 6:**
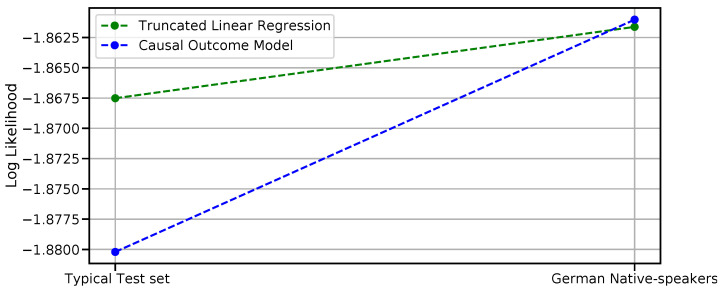
PLL for the Truncated Bayesian Linear Regression and the Causal Outcome Model on a “typical” test set (30% of randomly selected subjects) vs. a test set composed of uncommon subjects (i.e., German native speaking subjects).

Figure 6 depicts at a glance the comparison of the performance (average PLL) of the two best models, i.e., the purely associative predictive model (non-causal Truncated BLR) vs. the causal one (Causal Outcome Model) when testing on the “typical” and “uncommon” test sets (see, for reference, Table 5).

#### Discussion

*Causal factors*. The main findings can be condensed as follows. Higher agreement ratings are associated with a home base with higher valence. A similar result is obtained for both the dominance and engagement traits that are significantly related to a home base with higher arousal. Furthermore, higher variability in the arousal dimension ($D_{AA}$) is significantly linked to higher engagement traits.

Notably, when correcting for confounding, the regression coefficient associated with the home base valence dimension no longer produces statistically significant results when predicting for agreement. Conversely, the coefficients that presented a significant association between core affect dynamics and both dominance and engagement traits remain significant in the causal model. Technically, from a causal standpoint, these can be interpreted as the Average Treatment Effect (ATE) of observing a specific affective dynamics on the social trait attribution by a third-person observer. Specifically, results show that subjects exhibiting higher arousal will be rated as more dominant. Similarly, the level of engagement is positively affected by higher and more fluctuating arousal trajectories.

A first remark is that the Bayesian analysis of both approaches, correlational and causal, does not detect significant relationships between the V/A dynamics and the performance and rapport interpersonal behavioural traits. This result is not surprising for both traits given the oral instructions for annotation provided to the raters. The definition of the former (“the person’s speech is clear and relevant to the task”) only requires a kind of objective observation on verbal behaviour. The latter (“whether the person and his/her partner give the impression to be or they could become friends”) is likely to involve a complex judgement, where, if considered, either positive or negative values of V/A might be recruited depending on the rater’s individual cognitive evaluation process; this potential variability is also evident by considering the average ICC score for this factor in Table 4.

More interesting are the results achieved for the agreement, dominance and engagement traits. Note that, in terms of social-psychological models of social evaluation [81], the dominance trait is easily mapped on the general Agency dimension (represented as a bipolar axis ranging from assertive-dominant to passive- submissive behaviour), whilst agreement (“whether the subject seems to agree with his/her partner”) addresses the Communion dimension that is the bipolar axis ranging from agreeable to quarrelsome behaviour [82,83,84]. The fundamental dimension of agentic behaviour can be conceptualized as behaviour that asserts status relative to others; agency also refers to qualities relevant for goal attainment. Communal behaviour can be conceptualized as behaviour that promotes interpersonal ties [82,83,84]. Given the rating instructions (“the person shows to be engaged”) and the study context, engagement can also be mapped onto the Agency dimension as relevant for goal attainment (i.e., solving the survival task) [81].

Remarkably, under such circumstances, the model’s outcome is in accordance with previous psychological experimental results and theorizing that formulated specific hypotheses about how individual differences in intraindividual variability in core affect relate to those in interpersonal behaviour [52]. Precisely, experimental findings give evidence that the strongest association is achieved for arousal and agentic behaviour, whereas only a weak correspondence is measured between valence and the Communion dimension [52]. Interestingly, the analysis on the causal outcome rules out the mean valence dimension predicting for agreement as compared to the correlational outcome. In addition, the parameter associated with the diffusion (variability) in the arousal dimension is retained, in line with the assumption that variability in how dominant one behaves towards others is related to variability in how active one feels [52]. In spite of its essential and simple conceptual formulation, the phenomenological model behind the emulator overall captures well-known aspects concerning the relationship between affect and interpersonal behaviour.

*Causal Model Validation*. Notably, human and model-simulated raters exhibit excellent agreement (ICC>0.75); this quantity is comparable to the ICC value eventually obtained when comparing human raters only. Interestingly enough, multiple raters sampled from the outcome model exhibit a good agreement (0.6<ICC<0.74), thus revealing remarkable internal rating consistency.

Notice that the outcome of the measured ICC-based agreement, when human and model-simulated raters are considered together, provides for three fundamental social traits a higher score than those obtained from humans and model separately. This result is not surprising given that the perceived core affect trajectory is inferred starting from the labelling output of the EWE observer (Section 4.4.1), conforming to what is generally practiced in the affective computing realm.

*Causal Model Performance evaluation.* As can be appreciated by inspecting Table 5, the adopted baseline model (non-causal BLR) yields significantly lower performances if compared with the other two models on typical test sets. On the other hand, if comparing the PLLs delivered by the non-causal Truncated BLR with those obtained from the causal Outcome model on the same test set, the results are comparable. Indeed, albeit producing nonidentical performances (the causal model exhibiting slightly worse results), their difference is not significant (independent sample *t*-test, *p*-value > 0.05). Using the causal model does not worsen the prediction abilities with respect to the non-causal one. Indeed, on typical test sets, a good causal model should have prediction performances comparable to the non-causal one but should aim at producing better predictions when dealing with “uncommon” data, i.e., data under intervention (this feature is known as “stability”, “autonomy” or “modularity” [65]). This fact can be appreciated to some extent in Figure 6; when evaluating on a test set of uncommon data, the causal outcome model performance raises sensibly if compared with the associative model. Notably, on data under intervention, the relative performances of the two models is flipped, although—as for the typical test set case—with non statistically significant differences; however, the trend exhibited by the two models (Figure 6), besides the limits posed by the dimension of the test set, deserves to be reported.

Technically, the specific analysis employed here has some limitations that are worth mentioning. In particular, the emulator, in its present version, relies on the deconfounder method in order to perform causal inference from observational data. The original study presenting this approach [23] was deeply discussed in [69,85,86,87] and later refined in [88,89]. In these follow-up studies, several clarifications on the theoretical requirements of the deconfounder were presented. Besides the already discussed assumptions (no unobserved single cause confounders and no causal relationship between the causes), Ref. [89] adds a third requirement: the substitute confounders ($c_{j}$ with j=1,⋯,K) should be pinpointed by a a single deterministic function of the causes; in other words, the uniqueness of the factor model that captures the distribution of the causes is required. It has been shown [90,91] that, under certain conditions (many causes and low dimensional factor model latent space), inference of latent variables approaches a deterministic function. In our analysis, the factor model latent space dimension is kept very low (K=2) if compared with the number of causes; notably, the adopted Posterior Predictive Check procedure reveals a good capacity of the model to capture the joint distribution of the data. Nonetheless, it is worth remarking that the analysis is conducted on a limited number of data points, this quantity being constrained by the size of the adopted RECOLA dataset.

## 6. General Discussion

### 6.1. Findings and Implications

The emulator’s component based on the Ornstein–Uhlenbeck process finely captures (the perceived) core affect dynamics, in spite of its abstraction with respect to the complexity of the processes underpinning such psychological construct [32,33,34,35] and provides an effective input to the social judgement causal component. Model results are in line with previous studies for characterising individual’s core affect fluctuations and its bearing on interpersonal behaviour and personality [52,56,60]. The rationale behind this component is that core affect can be formally conceptualised in terms of a complex dynamical system [74,75,92]. This has led us to quantitatively assess model’s behaviour by introducing techniques borrowed from recurrence theory [75] that are, to the best of our knowledge, novel as to affect analysis. As such, this modelling choice has some general implications. On the one hand, it paves the way to the exploitation of the many linear and nonlinear time-series analyses techniques that can be employed to investigate the dynamics inherent to affective and social behavioural (time-series) data. On the other hand, to put this in perspective, tracking the change over time of its identifying parameters at a coarse-grained time-scale (e.g., in a longitudinal study) would allow for studying behaviour as a time-evolving phenomenon. This way, a principled understanding can be gained concerning the processes by which affective/social behaviours come about in day-to-day activities [74,93]. Interestingly enough, the first stage of the emulator could be exploited whenever an analysis in terms of stochastic trajectories is requested. One straightforward example is the analysis of gaze trajectories in social/affective behaviour [94,95].

In addition, it is worth recalling from the discussion in Section 4.4.1 the effect of the EWE-based inference of the perceived core affect trajectory impinging on the outcome of the measured ICC-based agreement (Table 4); namely, predictions over three social traits, when human and model-simulated raters were considered together, achieved higher ICC-agreement scores than those obtained from humans and model separately. Clearly, nothing prevents fitting the model on individual raters, which would provide at the study level (see, for reference, Figure 2) quantitative information concerning idiosyncrasies and consistency of the rater, markedly when rating over time is to be evaluated (which is not the case for the RECOLA dataset). In a machine learning perspective, this option could also serve the purpose of assessing normalization techniques of produced labelling and synthetic data generation, a cogent problem in current research [96].

As to the causal component of the emulator, all in all, the achieved results are in line with those established in the psychological literature that relate individual differences in intraindividual core affect dynamics within interpersonal behaviour [52]. This is a promising result considering that causal inference from observational data is a difficult task and requires strong assumptions. While causal inference usually considers a single possible cause, the deconfounder method [23] that we have adopted provides valid causal inference at least for the data we are handling here; this is achieved by exploiting the multiplicity of causes, which entails weaker assumptions than the classical approach requires. The unveiling of causal relationships can indeed improve the internal validity of the underlying study (which is curtailed when results can be explained by factors that are additional to those explicitly incorporated in the design) and subsequent machine learning analyses.

In this respect, notice that, for conceptual clarity, we have introduced the emulator as a specific subsystem functionally detached from both the dataset and the machine learning subsystems (see, for reference, Figure 2). However, when the chief concern is machine learning, the operationalisation of the emulator could entail its embedding within the machine learning component. A straightforward example can be constructed by adopting a computational model to derive core affect trajectories from multimodal data (e.g., [48] that has been experimented on the same dataset), which are then fed into the emulator to predict social traits. The simulation we have set up for assessing predictive performance shows promise but also reveals limitations that are discussed further below. However, in a stringent machine learning perspective, one might also consider adopting deep neural networks as causal estimators, a novel and vibrant research area [97,98]. Preliminary studies in this field claim, for low bias, suitability for estimating the heterogeneous treatment effects and providing opportunities to predict causal effects in untreated populations beyond the original sample. However, there is still little consensus on important practical considerations needed to deploy these tools in the wild [97]. For what concerns causal inference for affective/social behaviour, the long-term most promising avenue is offered by deep learning of the causal structure of dynamic systems and time series data [99]. Indeed, in real world cases, dependencies among time series are usually nonlinear and ignoring such interactions could lead to inconsistent estimation. However, using causal knowledge to improve machine learning algorithms remains an open area [98,100], and causal analysis in affective computing is at best in its infancy apart from a handful of exceptions (e.g., [100,101,102,103,104,105]).

However, theoretical knowledge of causality methods being combined with psychological observations [106] can provide interesting insights into the computational methods dealing with affect and social interaction.

Eventually, it is worth remarking that the methods we have introduced are general enough to be adopted in studies that do not specifically concern social participation. For instance, when dealing with self-esteem [107], one example might consider relations between indicators of self-esteem [107] and negative affect [108].

### 6.2. Limitations

Potential technical limitations concerning the deconfounder method per se have been discussed in Section 5.2. Furthermore, in the case considered in the present study, we had to face the hurdle of a slightly limited dataset—from a modern machine learning perspective—which reflects on the out-of-sample test case we have constructed. This limitation put constraints on the evaluation of causal analysis predictive performance. On the other hand, the emerging trend we have detected suggests that a causal approach is suitable to provide better predictions than a purely predictive model when uncommon test data are taken into account. Note that the term “uncommon” from the machine learning standpoint translates into the use of a novel dataset at the testing stage, which is nothing but the generalisation problem (addressing indeed the challenging setting where the testing distribution is unknown and different from the training, also called the out-of-sample or out-of-distribution setting), a cogent one in the field [109]. In this respect, it is acknowledged that causal inference is one key to cope with such problem [109]; in other terms, the causal approach can potentially pave the way to increase the external validity of a study. This is an open research problem in the field and the results we achieved here, though encouraging, do not allow for a conclusive statement in this respect.

### 6.3. Research Directions

In light of the above considerations, the suitability of the proposed methods to cope with the unfolding over time of affective/social behaviour raises the interesting point of whether they could prospectively be of service to experience sampling studies (also called ecological momentary assessment [110]); these have the virtue of examining behaviour in its natural, spontaneous context [111]. Experience sampling (ES) broadly denotes the set of empirical methods that are designed to allow respondents to document their thoughts, feelings, and actions outside the walls of a laboratory and within the context of everyday life. It is an idiographic approach addressing “within-person” patterns (thus concerning the intra-individual process), as opposed to the classic nomothetic approach (identifying patterns of behaviour across a population of individuals, rather than for any given individual) [112]. ES has gained currency for affective/social behavioural research [112] and is likely to play a role in the future of affective computing. On the one hand, currently available technologies have expanded the repertoire of ES techniques [113,114,115] offering novel possibilities; mobile devices are one such example [113,116,117]. Among others, devices that are likely to play a key role in the near future are smart mirrors [118,119]. Unfortunately, in most cases, researchers conducting experiments with novel devices adapted paper-and-pencil methods from classic ES sampling studies [114,117]. Furthermore, datamining large datasets generated by novel technologies is a promising methodology, which increases external validity (results can be generalized to groups other than those that participated in the original study) of results, given by the size of collected data [117]. Datamining, however, is challenged by the lack of datasets and the difficulty of aggregating user-generated data. Gathering data is costly, and labelling data more so. It is not surprising that ES is at best an underexplored approach in the affective computing field [1,2,3]. A handful of exceptions concern probing the user at suitable moments to collect self-reports via smartphone, e.g., [120]. In most advanced studies, the ES procedure is extended to exploit physiologically-triggered probing and recording of participants’ self-reports and peripheral physiological activity [121]. The adoption of devices such as smart mirrors [118,119], beyond the privacy concerns that we shall not address here, might offer the availability of multimodal data for subsequent analysis; in addition, these more complex interaction devices seem to have potential for longitudinal studies in the field under quasi-experimentation design [117] based on interval contingent triggering (e.g., interacting with the mirror twice a day) [116]. In the case of current ES studies, ratings to be evaluated are by and large self-reported; however, as long as we have time-series, the presented methods can be suitably adapted for gauging recurrent patterns and their variability together with indicators of affective/social states, namely that crystallise the bare essentials of ES [121]. Extending the device capabilities (e.g, smart mirrors) could allow for mixed ratings (personal and third-person).

The proposed approach offers the opportunity to face a number of problems in different realms of application. The assessment of displayed personality/social capabilities is important as they define the quality of social interactions. Notably, these are believed to positively contribute to health and well-being [122]. In this respect, mining the causes that result in a higher/lower level of perceived social participation may concur in understanding such phenomenon. Furthermore, automatic personality/social traits analysis tools are gaining more and more currency in affective and social computing applications whose primary concern is to produce artificial systems that are able to deal with social signals of people they interact with, and exhibit proper social behaviour as a response. As an example, social robots should be capable of displaying social competence in order to be perceived in a proper way. Notably, the way a robot is perceived by humans that it is supposed to interact with (e.g., its personality and social attitude) has been extensively studied in the social robotics field [123,124,125]. It seems therefore reasonable to argue that a social robot owning a causal model of its external social perception, equipped with the ability of simulating proper social behaviour, could be considered as more socially competent.

The final remark is a cautionary note. Under all the circumstances discussed above, it is worth noticing that many applications evolving from such tools might be potentially harmful [126]. Indeed, end-users are often unaware of the process leading to the final prediction and of the theoretical paradigms that the system implicitly accepts. As a result, they can easily make errors when interpreting the data. This risk should be minimized especially in sensitive fields where a misunderstanding of outcomes can lead to critical scenarios (e.g., automated job screening). To this end, models dealing with such kind of information should be able to provide actionable information; in other words, the model should be built in order to answer counterfactual questions [127]. In this regard, understanding the cause/effect relationships that elicit the attribution of apparent traits is of primary importance for the definition of trustworthy predictive models.

## 7. Conclusions

In this study, we have addressed a hitherto neglected problem. We have considered the typical scenario where raters are recruited to label (in continuous or discrete form) observed subjects in terms of their exhibited affective/social behavioural cues; in the case considered here, the rater is attributing both affective and social participation labels to the behaviour of subjects involved in an experiment concerning dyadic interactions under a given task. In spite of its widespread use in affective computing for constructing datasets, this setting most often conceals the occurrence of complex interactions between the rater, the subjects and the situational cues. As a matter of fact, the ground truth eventually achieved might be best conceived as the result of fragile equilibrium floating between many such factors, rather than the barely “objective” measure usually surmised. Thus, the results produced on its basis should be cautiously handled. Neglecting these issues can impinge on the internal validity of the underlying study and subsequent machine learning analyses.

To shed light on such complex interactions, we have proposed a novel model, a human rater emulator that relies upon a phenomenological representation of the core affect (the OU state-space model) coupled with a causal trait-based attribution of social attitudes.

As related to the specific research questions addressed in this work, the analyses conducted on the publicly available RECOLA corpus and results so far achieved allow for the following conclusions:

1. The core affect dynamics generated by the emulator is consistent with the participant’s affect dynamics as perceived by the human rater;
2. The phenomenological model behind the emulator overall reliably captures salient causal aspects concerning the relationship between core affect and interpersonal behaviour involved in social judgement;
3. The emulator in its present form, when straightforwardly embedded as a component of the machine learning pipeline, exhibits an interesting performance trend that is in line with the theoretical expectations, namely that a causal-based model should perform better than a correlational one in dealing with out-of-sample data. The difference in performance, however, that we have reported with the limited out-of-sample test set available does not allow for a conclusive statement in this regard, at least in terms of classical statistical significance.

The latter point calls for further investigation concerning the design of efficient causal models/architectures suitable for machine learning and the construction of corpora suitable to address the out-of-sample problem. Both problems are open issues in current research.

All in all, the emulator approach can be informative for both levels concerning the observational study/dataset and its machine learning exploitation, while prospectively offering the opportunity to face cogent problems in different realms of application.

## Figures and Tables

**Table 1:** Results of the Cross Recurrence Analysis (CRA) conducted among the V/A trajectories from human raters and 10 emulated raters for each of the 23 subjects. The table reports the mean values (±standard deviation) of three quantitative measures, namely Determinism (DET), Laminarity (LAM), and Maximum diagonal line length (MDL). The results of these measures are compared against those of 10 random raters.

Subject ID	DET		LAM		MDL	
	OU	Random	OU	Random	OU	Random
P16	0.965 ± 0.006	0.013 ± 0.005	0.965 ± 0.005	0.014 ± 0.005	67 ± 10	2 ± 0
P17	0.968 ± 0.005	0.006 ± 0.005	0.970 ± 0.004	0.006 ± 0.006	96 ± 39	2 ± 0
P19	0.969 ± 0.005	0.030 ± 0.020	0.968 ± 0.005	0.030 ± 0.020	78 ± 16	2 ± 0
P21	0.962 ± 0.003	0.018 ± 0.014	0.964 ± 0.003	0.018 ± 0.014	67 ± 12	2 ± 0
P23	0.967 ± 0.002	0.019 ± 0.012	0.968 ± 0.002	0.019 ± 0.012	93 ± 21	2 ± 0
P25	0.947 ± 0.005	0.016 ± 0.012	0.952 ± 0.005	0.016 ± 0.012	57 ± 11	2 ± 0
P26	0.968 ± 0.006	0.017 ± 0.017	0.968 ± 0.006	0.018 ± 0.017	81 ± 42	2 ± 0
P28	0.958 ± 0.003	0.017 ± 0.014	0.960 ± 0.003	0.017 ± 0.015	64 ± 10	2 ± 0
P30	0.955 ± 0.003	0.021 ± 0.012	0.958 ± 0.002	0.020 ± 0.012	68 ± 10	2 ± 0
P34	0.964 ± 0.003	0.016 ± 0.013	0.966 ± 0.002	0.015 ± 0.013	70 ± 8	2 ± 0
P37	0.965 ± 0.002	0.011 ± 0.011	0.965 ± 0.002	0.010 ± 0.011	59 ± 6	2 ± 0
P39	0.968 ± 0.001	0.022 ± 0.020	0.968 ± 0.001	0.022 ± 0.021	74 ± 10	2 ± 0
P41	0.973 ± 0.002	0.018 ± 0.022	0.973 ± 0.002	0.018 ± 0.022	111 ± 22	2 ± 0
P42	0.961 ± 0.003	0.013 ± 0.011	0.962 ± 0.002	0.012 ± 0.011	61 ± 19	2 ± 0
P43	0.964 ± 0.003	0.011 ± 0.007	0.964 ± 0.002	0.010 ± 0.008	68 ± 13	2 ± 0
P45	0.952 ± 0.004	0.019 ± 0.014	0.957 ± 0.003	0.019 ± 0.015	58 ± 12	2 ± 0
P46	0.945 ± 0.005	0.022 ± 0.013	0.949 ± 0.004	0.022 ± 0.014	52 ± 7	2 ± 0
P48	0.956 ± 0.004	0.018 ± 0.015	0.959 ± 0.004	0.018 ± 0.015	64 ± 8	2 ± 0
P56	0.968 ± 0.003	0.023 ± 0.016	0.968 ± 0.003	0.023 ± 0.016	79 ± 19	2 ± 0
P58	0.960 ± 0.003	0.010 ± 0.006	0.960 ± 0.002	0.010 ± 0.006	57 ± 7	2 ± 0
P62	0.968 ± 0.002	0.013 ± 0.013	0.968 ± 0.002	0.014 ± 0.013	80 ± 13	2 ± 0
P64	0.961 ± 0.004	0.016 ± 0.016	0.963 ± 0.003	0.016 ± 0.016	76 ± 26	2 ± 0
P65	0.960 ± 0.003	0.013 ± 0.009	0.960 ± 0.002	0.012 ± 0.009	69 ± 18	2 ± 0

**Table 2:** Bayesian Linear Regression model coefficients not containing zero value in the HDI of the posterior.

Coefficient	Description	Mean Post. Distrib.	95% HDI
Agreement			
$\beta_{Agr}\left[U_{V}\right]$	η associated with the home base for the Valence dimension	0.54	0.056÷1.03
Dominance			
$\beta_{Dom}\left[U_{A}\right]$	η associated with the home base for the Arousal dimension	0.56	0.14÷0.97
Engagement			
$\beta_{Eng}\left[U_{A}\right]$	η associated with the home base for the Arousal dimension	0.62	0.27÷0.98
$\beta_{Eng}\left[D_{AA}\right]$	η associated with the diffusion on the Arousal dimension	0.44	0.13÷0.73

**Table 3:** Causal Bayesian Linear Regression model coefficients not containing zero value in the HDI of the posterior.

Coefficient	Description	Mean Post. Distrib.	95% HDI
Dominance			
$\eta_{Dom}\left[U_{A}\right]$	η associated with the home base for the Arousal dimension	0.54	0.076÷1
Engagement			
$\eta_{Eng}\left[U_{A}\right]$	η associated with the home base for the Arousal dimension	0.57	0.17÷0.97
$\eta_{Eng}\left[D_{AA}\right]$	η associated with the diffusion on the Arousal dimension	0.49	0.17÷0.84

**Table 4:** Intra-class correlation (ICC) measuring agreement between 6 human raters (ICC Humans), 6 raters sampled from the outcome model (ICC Model) and the 12 human + model simulated raters (p<0.05 for all cases).

	ICC Humans	ICC Model	ICC Humans + Model
agreement	0.72	0.72	0.82
dominance	0.75	0.74	0.79
engagement	0.82	0.75	0.85
performance	0.83	0.73	0.79
rapport	0.77	0.63	0.62
average	0.78	0.72	0.77

**Table 5:** Average Predictive Log Likelihood and standard deviation for non-causal Vanilla Bayesian Linear Regression, non-Causal Truncated Bayesian Linear Regression and causal Outcome Model on a typical test set and on a test set of uncommon subjects (German native speakers).

	Non-Causal BLR	Non-Causal Trunc. BLR	Causal Outcome Model
Typical Test	−3.07±0.19	−1.87±0.01	−1.88±0.008
Uncommon Test	−1.94±0.13	−1.862±0.009	−1.861±0.01

## Data Availability

Data for reproducing the experiments reported in this article are available at this https://diuf.unifr.ch/main/diva/recola (accessed on 28 December 2022).

## Abbreviations

The following abbreviations are used in this manuscript:

OU	Ornstein–Uhlenbeck
OU-SSM	Ornstein–Uhlenbeck State-Space Model
BLR	Bayesian Linear Regression
ADVI	Automatic Differentiation Variational Inference
ATE	Average Treatment Effect
SUTVA	Stable Unit Treatment Values Assumption
IRR	Inter-Rater Reliability
ICC	Intra-Class Correlation
RA	Recurrence Analysis
CRA	Cross-Recurrence Analysis
CRP	Cross-Recurrence Plot
DET	Determinism
LAM	Laminarity
MDL	Maximum diagonal line length
PPCA	Probabilistic Principal Component Analysis
PPC	Posterior Predictive Checks
PLL	average Predictive Log-Likelihood
HDI	Highest Density Interval
ES	Experience Sampling
